# Tri-State Medical Association Call

**Published:** 1889-10

**Authors:** Frank Trester Smith

**Affiliations:** Sec’y of Committee; Chattanooga, Tenn.


Chattanooga, Tenn., August 9, 1880.

DEAR DOCTOR:

The following call has been issued:

"The members of the medical profession in Alabama, Georgia and Tennessee are requested to meet in Chattanooga on the Third Tuesday in October, for the purpose of forming a Tri-State Medical Association. All will be admitted to the meeting of the Association, but the membership will be restricted to graduates of regular Medical Colleges in good standing."

This call is signed by committees from Jackson County, Alabama, Medical Society; Chattanooga, Tennessee, Medical Society; Cleveland, Tennessee, Medical Society; Cartersville, Georgia, Medical Society; Dalton, Georgia, Medical Society.

Fraternally yours,

FRANK TRESTER SMITH, M. D.,

Sec'y of Committee.

